# Synthesis of Fe_16_N_2_ compound Free-Standing Foils with 20 MGOe Magnetic Energy Product by Nitrogen Ion-Implantation

**DOI:** 10.1038/srep25436

**Published:** 2016-05-05

**Authors:** Yanfeng Jiang, Md Al Mehedi, Engang Fu, Yongqiang Wang, Lawrence F. Allard, Jian-Ping Wang

**Affiliations:** 1 Department of Electrical and Computer Engineering, University of Minnesota, Minneapolis, MN 55455 USA; 2 Department of Chemical Engineering and Materials Science, University of Minnesota, Minneapolis, MN 55455 USA; 3 Ion Beam Material Laboratory, Los Alamos National Laboratory, Los Alamos, New Mexico 87545 USA; 4 Materials Science and Technology Division, Oak Ridge National Laboratory, Tennessee 37831, USA

## Abstract

Rare-earth-free magnets are highly demanded by clean and renewable energy industries because of the supply constraints and environmental issues. A promising permanent magnet should possess high remanent magnetic flux density (B_r_), large coercivity (H_c_) and hence large maximum magnetic energy product ((BH)_max_). Fe_16_N_2_ has been emerging as one of promising candidates because of the redundancy of Fe and N on the earth, its large magnetocrystalline anisotropy (Ku > 1.0 × 10^7^ erg/cc), and large saturation magnetization (4πMs > 2.4 T). However, there is no report on the formation of Fe_16_N_2_ magnet with high B_r_ and large H_c_ in bulk format before. In this paper, we successfully synthesize free-standing Fe_16_N_2_ foils with a coercivity of up to 1910 Oe and a magnetic energy product of up to 20 MGOe at room temperature. Nitrogen ion implantation is used as an alternative nitriding approach with the benefit of tunable implantation energy and fluence. An integrated synthesis technique is developed, including a direct foil-substrate bonding step, an ion implantation step and a two-step post-annealing process. With the tunable capability of the ion implantation fluence and energy, a microstructure with grain size 25–30 nm is constructed on the FeN foil sample with the implantation fluence of 5 × 10^17^/cm^2^.

Rare-earth-free magnets are highly demanded by clean and renewable energy industries because of the supply constraints and environmental issues of rare-earth permanent magnets in recent years^1^. Among many candidates being pursued^1^,^2^, Fe_16_N_2_ has been emerging as one of promising candidates because of the redundancy of Fe and N on the earth, its large magnetocrystalline anisotropy (K_u_ > 1.0 × 10^7^ erg/cc), and large saturation magnetization (4πM_s_ > 2.4 T)^3^. A promising permanent magnet should have high Ms, large coercivity (Hc) and hence, large energy product. In this sense, α˝-Fe_16_N_2_ could be a promising permanent magnet^3^,^4^. Its theoretical energy product is estimated as high as 135 MGOe, corresponding to a fully packed single domain microstructure^3^. Many research groups have investigated α˝- Fe_16_N_2_ during the last 40 years^2^,^3^,^5^,^6^,^7^,^8^,^9^,^10^, while attempting many preparation methods. However, there is no report on the formation of Fe_16_N_2_ magnet with high B_r_ and large H_c_ in bulk format yet. Based on previous experience in α˝-Fe_16_N_2_ thin film preparation, suitable nitrogen concentration (11.1 at.%) is necessary for a high volume ratio of α˝-Fe_16_N_2_ phase^3^,^11^. At the same time, residual strain is also one of the key aspects needed to induce martensite phase formation and achieve giant Ms^12^. Moreover, its microstructure, including grain size and grain boundary, should be optimized at the same time to achieve high coercivity^13^. So, nitriding, strain and microstructure are the three important aspects of Fe_16_N_2_ permanent magnet preparation.

So far, no approach is reported to prepare Fe_16_N_2_ bulk samples, which could optimize all these three key aspects, including nitriding, strain and microstructure, at the same time. We suspect that some contradictions are aroused by the discrepancy between inherent material properties and required technical parameters. For example, the microstructure of a permanent magnet can be adjusted during the post-annealing process by changing the annealing temperature^13^. For Fe-based material, its microstructure can be tuned when the annealing temperature above 300 °C^14^. However, α˝-Fe_16_N_2_ is a martensite phase and can only be stable under 214 °C^2^. This contradiction means the microstructure of α˝-Fe_16_N_2_ can’t be directly tuned by traditional annealing method. This is why, until now, there isn’t any report on how to tune the microstructure on α˝-Fe_16_N_2_ bulk sample yet.

Table 1 shows the current technical status on these three aspects^2^,^3^. For thin film samples, the lattice mismatch acting as a strain source plays an important role in the phase transformation^3^,^4^,^15^ and giant Ms could be observed on thin film samples. While for powder samples, no strain is applied and the corresponding Ms is always lower than Fe^8^,^9^,^10^. No result has been published on how to construct a suitable microstructure in α˝-Fe_16_N_2_ thin film and powder. So, for the thin film sample and the powder sample with large particle size (>50 nm), partial soft magnetic property is always observed. While the powder samples with smaller particle size (<50 nm), were reported to exhibit higher coercivity^8^,^9^,^10^ because the particle size is coincidental in the range of required grain size for high coercivity^13^.

To prepare an α˝-Fe_16_N_2_ permanent magnet in bulk format, it is strategically important to investigate one synthesis technique, by which nitriding, strain and microstructure engineering could be all taken into consideration.

Here we use a nitrogen implantation technique to prepare bulk α˝-Fe_16_N_2_ with hard magnetic property. Nitrogen ion implantation is considered as an alternative nitriding approach with the benefit of tunable implantation energy and fluence. Ion-implantation methods can be considered as an approach to prepare bulk α˝-Fe_16_N_2_ samples since it is a commercially established technology in the semiconductor industry. By this method, the nitrogen concentration and penetrable depth can be controlled precisely. This technique was used by other groups to prepare Fe_16_N_2_ thin film^6^,^7^,^16^,^17^ and its feasibility was demonstrated by tuning the implantation energy and fluence. However, there wasn’t any reported coercivity on the implanted samples. Furthermore, it was found that the α˝-Fe_16_N_2_ phase disappeared when the thickness was over 230 nm^18^, possibly caused by the fact that the α˝-Fe_16_N_2_ phase was unstable under high ion fluence and transformed into ε nitride phase. These problems hindered the application of this technique on bulk α˝-Fe_16_N_2_ preparation.

The main technical strategy involves nitrogen implantation into free-standing iron foils and transformation into Fe_16_N_2_ phase assisted by the thermal strain during the annealing process. By tuning the fluence, a suitable microstructure can be obtained^19^, corresponding to high coercivity, and hence hard magnetic property.

Nakajima *et al.* for first time used the nitrogen ion implantation process to prepare Fe_16_N_2_ thin film deposited on a substrate^7^,^20^, but the Fe_16_N_2_ layer is neither free-standing material nor possessing any hard magnetic property, e.g. very low coercivity. In this paper, we successfully synthesize free-standing Fe_16_N_2_ foils with a coercivity of up to 1910 Oe and a magnetic energy product of up to 20 MGOe at room temperature. An integrated synthesis technique is developed, including a direct foil-substrate bonding step, an ion implantation step and a two-step post-annealing process. With the tunable capability of the ion implantation fluence and energy, a microstructure with grain size 25–30 nm is constructed in the FeN foil sample with the implantation fluence of 5 × 10^17^/cm^2^.

Figure 1 shows the relevant characterization results of a sample with 5 × 10^17^/cm^2^ fluence. A hard magnetic property with high coercivity (1910 Oe), high Ms (245 emu/g), and promising energy product (Max. 20 MGoe) is clearly observed on the sample. Figure 1(a) shows in-plane hysteresis loops for the sample at room temperature. There is an obvious shoulder at low field, indicating two different magnetic phases existing in the sample and coupling through the exchange-spring effect^21^. The direct calculated energy product is shown in Fig. 1(b), showing its maximum value to be 20 MGOe. The X-ray diffraction spectrum of the sample, as shown in Fig. 1(c), indicates a partial Fe_16_N_2_ phase coexisting with Fe and Fe_4_N phases. The crystalline structure is demonstrated by the transmission electron microscopy (TEM) diffraction pattern, as shown in Fig. 1(d).

## Results

Figure 2 shows room temperature M-H loops for the sample with different fluence at 100 keV implantation energy. The characterization is conducted in the foil plane using a Vibrating Sample Magnetometer (VSM) calibrated by a standard Ni sample at room temperature. Compared to 206 emu/g for the control sample (the starting single crystal iron foil), four samples, with fluences of 2 × 10^16^/cm^2^, 8 × 10^16^/cm^2^, 1 × 10^17^/cm^2^ and 5 × 10^17^/cm^2^, exhibit high Ms, varying from 230 emu/g to 245 emu/g with different volume ratio of Fe_16_N_2_ phase. For the sample with 1 × 10^18^/cm^2^ fluence, its Ms value drops to 208 emu/g because of the absence of Fe_16_N_2_ phase with high fluence.

To get a clear picture on the influence of the formed Fe_16_N_2_ phase on magnetic properties of the samples, Figure S5 in supplementary section shows the hysteresis loops before ion implantation and before post-annealing. As shown in Fig. S5, for the pure iron foil before ion-implantation, its magnetic property is in good agreement with Fe (110) single crystal (loop a). Its remanent magnetization value is equal to its saturation magnetization value, which is around 206 emu/g at room temperature. For the sample after the ion-implantation and 500 °C × 0.5 hr pre-annealing step (loop b), its saturation magnetization increases about 7%, up to 221 emu/g. Meanwhile, its remanent magnetization value is reduced and its saturation field (Hs) is enhanced up to about 1000 Oe, which indicates the existence of the Fe_8_N phase after the pre-annealing step.

The successive post-annealing step at 150 ^o^C for 40 hrs tremendously changes the M-H loop of the sample, which matches well with the formation of the Fe_16_N_2_ phase in the sample as indicated in Fig. 2. As shown by hysteresis loops in Fig. 2, a hard magnetic behavior is clearly observed with its saturation field up to 3.8 kOe. This is consistent with the existence of large magnetocrystalline anisotropy due to the body-center-tetragonal (bct) structure in Fe_16_N_2_^3^,^22^,^23^. More importantly, a 15% increase in the saturation magnetization is observed in this sample, which is much beyond the VSM testing error. The absolute Ms value is up to 245 emu/g under 5 × 10^17^/cm^2^ fluence, compared to 206 emu/g for the control sample (the starting single crystal iron foil). XRD patterns as shown in Fig. S3 and TEM analysis shown in Fig. S4 present clearly the mixed phases of Fe and Fe_16_N_2_ in the sample. Based on an XPS method^24^, the estimated volume ratio for the Fe_16_N_2_ phase in this sample under 5 × 10^17^/cm^2^ fluence is about 35%. Thus, the saturation magnetization of Fe_16_N_2_ phase in this sample is calculated as about 296 emu/g (2.9 T). For this calculation, we assume that the Fe matrix in the sample possesses the same saturation magnetization before the ion-implantation and annealing, which is reasonable, since we used the same sample before and after ion-implantation. This giant saturation magnetization is consistent with the proposed theory^15^ and recent report on thin film samples^3^,^12^. For sample with fluence 1 × 10^18^/cm^2^, the Fe_16_N_2_ phase decreases while the γ’-Fe_4_N/Fe_4−x_Ni_x_N phase increases, showing that Fe_16_N_2_ is unstable at higher fluence and decomposes into γ’-Fe_4_N/Fe_4−x_Ni_x_N phase.

**Figure 1:**
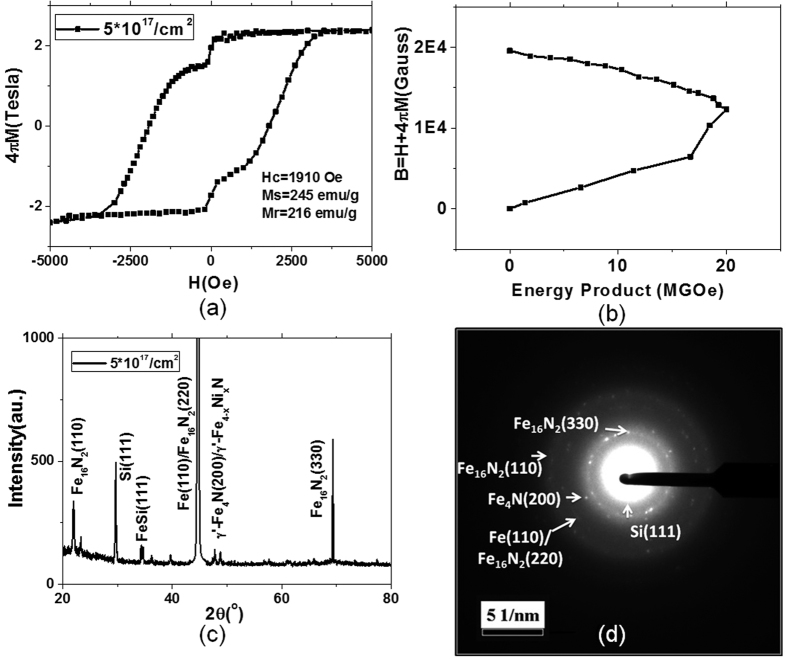
Characterization results of a bulk Fe_16_N_2_ free-standing foil prepared by the nitrogen ion implantation method with 5 × 10^17^/cm^2^ fluence (**a**) In-plane hysteresis loops for the sample at room temperature, showing Hc = 1910 Oe, Ms = 245 emu/g, Mr = 216 emu/g; (**b**) The calculated energy product, indicating maximum value 20 MGOe; (**c**) The X-ray diffraction spectrum, showing the Fe_16_N_2_ phase generated in the foil; (**d**) The HRTEM diffraction pattern of FeN sample with 5 × 10^17^/cm^2^ fluence, showing Fe_16_N_2_, Fe_4_N/Fe_4−x_Ni_x_N and Fe phases.

As shown in Fig. 1(c), FeSi (111) phase is generated. This indicates that Fe foil may be mixed with silicon substrate top surface during the sample preparation because of a lower surface energy of Si compared to that of Fe. Surface energy is one of the most fundamental parameters of a solid since it depends directly on the binding forces of the material. Indeed, it is a measure of the work necessary to separate a material into two parts along a plane. The surface energy of Si (111)^25^ is 1230 ergs/cm^2^ while the surface energy of Fe (110)^26^ is 2500 ergs/cm^2^. During the bonding process at 450 °C, silicon atoms could be cleaved and diffuse into Fe foil.

It is possible to produce Fe_4−x_Ni_x_N during annealing accompanied with the generation of Fe_4_N, in which Ni atoms occupy the FCC positions (Fe^f^) of the Fe_4_N in a random manner^27^. While the Ni atoms in Fe_4−x_Ni_x_N lattice carry zero magnetic moment, the magnetic moment in Fe_4−x_Ni_x_N is decreased accordingly. While high Ms value is observed in the implanted sample, it means that the volume ratio of Fe_4−x_Ni_x_N is not high enough to cancel the contribution of α˝-Fe_16_N_2_ phase. For the sample with fluence 5 × 10^17^/cm^2^, only 1% to 2% of Fe_4_N + Fe_4−x_Ni_x_N exists in the sample. The influence of magnetic moment degradation of Fe_4−x_Ni_x_N can be neglected. But for the sample with fluence 1 × 10^18^/cm^2^, volume ratio of Fe_4_N + Fe_4−x_Ni_x_N increases to 5% to 6%. The reason for the degradation of the Ms value of the sample should come from two aspects: decrement of the α˝-Fe_16_N_2_ phase and increment of Fe_4−x_Ni_x_N phase. To obtain sample with high Ms value, Fe_4−x_Ni_x_N phase should be avoided in future experiment.

**Figure 2:**
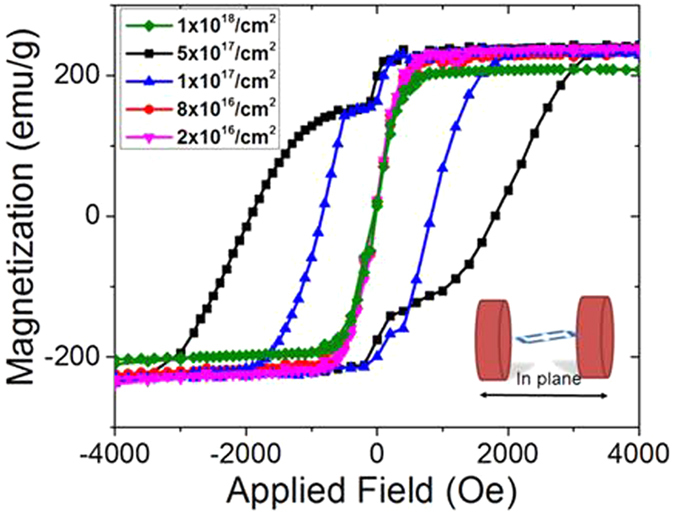
In-plane hysteresis loops for the samples with different fluences at room temperature. Samples with 2 × 10^16^/cm^2^, 8 × 10^16^/cm^2^ and 1 × 10^18^/cm^2^ fluences show soft magnetic property, while samples with 1 × 10^17^/cm^2^ and 5 × 10^17^/cm^2^ exhibit hard magnetic property.

For samples with fluences of 1 × 10^17^/cm^2^ and 5 × 10^17^/cm^2^, obvious flatness in the hysteresis loops can be observed, as shown in Fig. 2, identical to the behavior demonstrated for the exchange-spring magnets^21^. Another obvious evidence for the exchange coupling is that the ratio Mr/Ms in the well-processed samples is greater than 0.8^28^.

## Discussion

We experimentally demonstrate an integrated synthesis method to prepare α˝-Fe_16_N_2_ permanent magnets based on free-standing Fe foil samples with a thickness of 500 nm. The three key aspects for α˝-Fe_16_N_2_ permanent magnet preparation, including nitriding, strain and microstructure engineering, are addressed simultaneously in this method.

Nitrogen is introduced directly by the implantation method. For our experiment, the beam current density is 4 μA/cm^2^. The target temperature was intended at room temperature. The beam heating causes some temperature rise but usually no more than 50 °C since the active cooling on the sample stage was used. Generally the heat generated by high energy ion implantation would release the nitrogen atoms and also cause the phase transformation. The usage of high energy ion implantation above 100 keV was not possible to obtain Fe_16_N_2_^17^. Ion beam voltage has to be below 100 keV and current should be low to keep foil at low temperature. It was demonstrated experimentally that N + ion implantation with ion beam voltage of no more than 100 keV and current less than 5 μA was effective for Fe_16_N_2_ formation^17^,^29^. On the other side, low implantation energy (less than 100 keV) is also not good for α˝-Fe_16_N_2_ phase formation because of the requirement from the enthalpy of formation of α˝-Fe_16_N_2_^28^. The implantation energy determines the penetration depth directly. To get homogeneous distribution of nitrogen inside sample, a long term post-annealing is always required. If implantation energy is less than 100 keV, the post-annealing time will be prolonged correspondingly. A tradeoff between a reasonable annealing time and a good penetration depth should be considered. In this sense, the best implantation condition in this study is 100 keV.

To prevent nitrogen from escaping during annealing, a nickel layer was deposited on the surface of the foil^30^. Nickel cover layer is helpful in keeping nitrogen atoms inside. This is also helpful for nitrogen homogeneous distribution. To clean the absorbed oxygen on the iron surface, a surface reduction step was conducted before nickel deposition with 10% H_2_ + 90% N_2_ at 200 °C for 10 mins.

For the implanted nitrogen atoms, they are inert and deactivated before activation treatment. The pre-annealing step acts as an activation process to activate the implanted nitrogen. Temperature was increased to 500 °C to initiate the activation and remained at 500 °C for 0.5 hr to assist the activation over the entire wafer. Besides the function for activation, the pre-annealing step also helped to repair the lattice damage at 500 °C in Ar.

The strain is generated during the post-annealing process because of the mismatch of thermal coefficients between Fe foil and Fe substrate. Figure S6 in supporting material shows the strain by comparing XRD spectrum before and after annealing.

As for the microstructure, implanted ions with suitable fluence and energy can lead to specific grain size in the sample^19^.

Before post-annealing, nitrogen atoms are arranged in a disordered manner. The phase transformation into α˝ corresponds to a rearrangement of nitrogen atoms, resulting in an ordered pattern. Therefore, the post-annealing step assists with the ordering of the nitrogen atoms.

The other benefit of the long annealing time is the mechanical equilibrium of strain in foil. The thermal expansion coefficients of the iron foil and silicon substrate are 11.8 μm/m∙K and 2.6 μm/m∙K, respectively. The difference in the coefficients of linear thermal expansion between the iron and silicon will lead to the compressive stress on the iron foil. This difference Δα(T) is nearly constant between room temperature (RT) and the annealing temperature 150 °C. If the annealing is in mechanical equilibrium, that is, if the tension strain is commensurate with the Si substrate, a compressive strain will develop in the foil^31^:


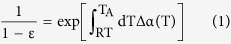


where ε denotes the strain and T_A_ is the annealing temperature.

The calculated linear compressive strain at 150 °C annealing is about 0.2%. A long annealing time is needed to produce strained foil in mechanical equilibrium.

In this way, a stretching force is generated along <001> crystalline direction in the foil with (110) face, which is responsible for the phase transformation from bcc to bct^3^. XRD spectrum in Fig. S2 shows that Fe_16_N_2_ phase appeared after the annealing process, which confirms the influence of the stress on the Fe_16_N_2_ phase formation.

The third aspect necessary for Fe_16_N_2_ permanent magnet is the microstructure engineering, which is directly related with the coercivity. Here it is found that microstructure is influenced by implantation dose at fixed implantation energy.

**Figure 3:**
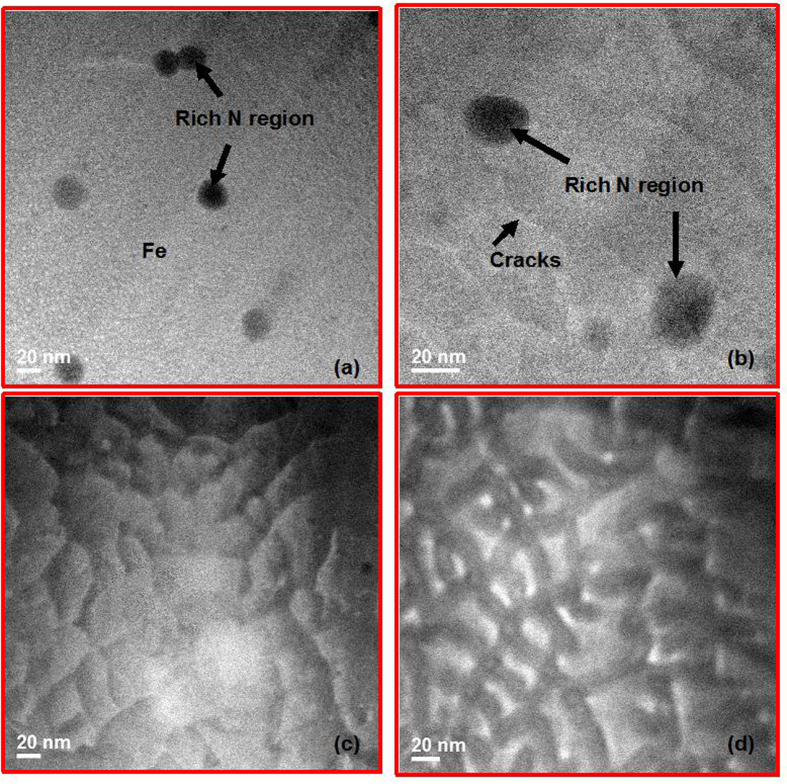
Microstructures of the prepared samples, observed by high resolution transmission electron microscopy (HRTEM). (**a**) 2 × 10^16^/cm^2^ fluence, embedded dots can be observed (rich N region), with a diameter of 20 nm, separated by 140 to 200 nm; (**b**) 8 × 10^16^/cm^2^ fluence, besides embedded dots, obvious cracks appeared; (**c**) 1 × 10^17^/cm^2^ fluence, an obvious microstructure is generated, with diameter 20 to 40 nm; (**d**) 5 × 10^17^/cm^2^ fluence, a microstructure with clear boundary is generated.

The coercivity is related to its microstructural profile, as shown in Fig. 3. The TEM sample is obtained by cutting and polishing the foil perpendicular to the surface using a Focused Ion Beam (FIB). It can be seen that there exists an obvious difference in the microstructure. For the four samples, all processes are the same except for variations in the fluences. It can therefore be deduced that the different implanted fluencies are the main reason for the development of a microstructure, and hence the coercivity variations.

Generally, a granular structure, instead of a homogeneous one, is formed on these samples, as shown in Fig. 3. At low fluences (2 × 10^16^/cm^2^ and 8 × 10^16^/cm^2^), the microstructure is a clear surface embedded by multiple black dots with diameters around 20 nm, while the distance from each other is 140 nm to 200 nm. The corresponding FFT diffraction shows the black dots are mixture phase of Fe_16_N_2_ phase and Fe. For medium fluences (1 × 10^17^/cm^2^ and 5 × 10^17^/cm^2^), continuous grains have been generated. For the 5 × 10^17^/cm^2^ sample (Fig. 3(d)), a much more obvious boundary can be observed.

The granular structure formation can be explained using the stochastic model^19^, accounting for the fluence dependence of the grain size. In this model, three mechanisms were presumed to account for the final grain size: statistical variations of area coverage by the implanted ions, ion channeling, and spontaneous nucleation^19^. The ions do not uniformly cover the surface. They strike the target surface at random points. So, for samples with low fluences, such as 2 × 10^16^/cm^2^ and 8 × 10^16^/cm^2^, some areas will appear nitrogen rich, as shown in Fig. 3(a,b). While at higher fluences, a more homogeneous distribution of implanted nitrogen appears, as shown in Fig. 3(c,d).

In case of medium mass ions of energy in the 100 keV range, the typical defects formed are void-like vacancy clusters composed of few vacancies^18^. For the situation of nitrogen implantation in this paper, such clusters act as traps for impurity atoms at low implantation doses and are progressively transformed into precipitates of new phases when the concentration of the impurity atoms becomes sufficiently high. The presence of these traps is crucial for nitride formation. During post-annealing, the short-range migration of nitrogen atoms toward the impurity atoms precipitates new phases and, consequently, the initially uniform distribution of implanted atoms transforms into a ˝granular˝ structure of impurity-rich precipitates embedded in an impurity-poor matrix. This can also be demonstrated by the hysteresis loop shown in Fig. 2. There is an obvious shoulder in the loop, showing two magnetic phases with different coercivity and Ms.

Based on the stochastic model, we have developed a numerical relationship to account for the variation of grain size with the implanted nitrogen fluence. The percentage of grains is determined from the statistics of the impact positions of the ions. As for samples with fluences of 1 × 10^17^/cm^2^and 5 × 10^17^/cm^2^, the calculated grain size is 2.5 × 10^−11^cm^2^. If the grain is assumed to be round, the grain length is 28 nm. This result fits very well with the experimental data shown in Fig. 3(c,d).

## Conclusions

An integrated technique that can be used for the preparation of α˝-Fe_16_N_2_ compound permanent magnets was proposed and demonstrated based on an ion implantation technology. In this approach, nitrogen ion implantation, direct bonding and a two-step annealing process were integrated for Fe_16_N_2_ permanent magnet preparation. First, free-standing iron foil was bonded directly onto a silicon substrate. An ion implantation process was used to obtain an Fe-N mixture in iron foils. Different nitrogen implant fluences with the same implant energy were investigated. A pre-annealing step at 500 °C for 0.5 hour was proposed and found to be crucial for three functions: (1) activating implanted nitrogen ions; (2) repairing the lattice damage; and (3) cleaning the sample surface. A post-annealing step at 150 °C for 40 hours was found to assist nitrogen atom diffusion and form the chemically ordered Fe_16_N_2_ phase in iron foils.

The M-H loop of a sample with 5 × 10^17^/cm^2^ fluence showed explicit improvement of magnetic properties, especially showing magnetic hard behavior after the post-annealing step. To the best of our knowledge, this could be the first experimental evidence of the existence of a giant saturation magnetization, an obviously large coercivity with a magnetic energy product of up to 20 MGOe in a bulk-type FeN sample. A granular structure was observed in the sample, in which a microstructure with 25–30 nm grains and obvious grain boundaries occurs.

## Experiment method

Pure (110) iron foils with 500 nm thickness are positioned on mirror-polished (111) Si substrate. The surfaces of the substrates and iron foils are cleaned beforehand. The foils are directly bonded with the substrate using a wafer bonder in fusion mode (SB6, Karl Suss Wafer Bonder) at 450 °C for 30 minutes.

Nitrogen ion implantation was conducted at the Los Alamos National Laboratory. Ions of atomic N^+^ were accelerated to 100 keV and implanted into foils vertically with fluences ranging from 2 × 10^16^/cm^2^ to 1 × 10^18^/cm^2^ at room temperature. After that, a two-step post-annealing process was applied on the implanted foils. The first step was pre-annealing at 500 °C in N_2_ and Ar mixed atmosphere for 0.5 hour. Post-annealing followed at 150 °C for 40 hours in a vacuum.

## Additional Information

**How to cite this article**: Jiang, Y. *et al.* Synthesis of Fe_16_N_2_ compound Free-Standing Foils with 20 MGOe Magnetic Energy Product by Nitrogen Ion-Implantation. *Sci. Rep.* **6**, 25436; doi: 10.1038/srep25436 (2016).

## Supplementary Material

Supplementary Dataset 1

## Figures and Tables

**Table 1:** Technical status of,and main obstructions to, permanent magnet preparation using thin film or powder^3^,^4^,^8^,^9^,^10^,^12^,^22^.

	Nitride	Strain	Microstructure	Magnetic property
Thin film	Plasma nitriding during deposition	Strain by lattice mismatch between FeN layer and substrate (Giant Ms)	No action (Low coercivity)	soft magnetic property
Powder	Nitriding in wide range temperatures (150 °C to 650 °C)	No strain (No giant Ms)	No action (Coercivity depends on its size)	Soft magnetic property on larger particles, hard magnetic property on small particle (<50 nm)
