# Supplementary material for: Synthesis of Fe16N2 compound Free-Standing Foils with 20 MGOe Magnetic Energy Product by Nitrogen Ion-Implantation
Source: Sci Rep. 2016 May 5;6:25436. doi: 10.1038/srep25436 (PMC4857173; doi:10.1038/srep25436)
Supplement: Supplementary Dataset 1 [file srep25436-s1.doc]

# **Synthesis of Fe_16_N_2_ compound Free-Standing Foils with 20 MGOe Magnetic Energy Product by Nitrogen Ion-Implantation**

Yanfeng Jiang^1)^, Md Al Mehedi^2)^, Engang Fu ^3)^, Yongqiang Wang^3)^, Lawarence F. Allard^4)^, and Jian-Ping Wang^1,2.*^

^1^Department of Electrical and Computer Engineering, University of Minnesota, Minneapolis, MN 55455 USA

^2^Department of Chemical Engineering and Materials Science, University of Minnesota, Minneapolis, MN 55455 USA

^3^ Ion Beam Material Laboratory, Los Alamos National Laboratory, Los Alamos, New Mexico 87545 USA

^4^Materials Science and Technology Division, Oak Ridge National Laboratory, Tennessee 37831, USA

*Corresponding author, E-mail: jpwang@umn.edu

***Supporting online material***

Experimental Methods

Figure S1 shows the integrated technical process to prepare α˝-Fe_16_N_2_ free-standing foils.

Here, a method to generate the stress in iron foil is designed to induce the crystalline deformation. Direct bonding technology[^1^](#_ENREF_1) is adopted during the pre-annealing process to bond the iron foil onto a silicon substrate. The surfaces of the substrates and iron foils are cleaned beforehand. The foils are directly bonded with the substrate by using a wafer bonder in fusion mode (SB6, Karl Suss Wafer Bonder) at 450 °C for 30 minutes. After the nitrogen ion implantation, a post-annealing process generates strain inside the foil by utilizing differences in thermal expansion coefficients between iron foil and silicon substrate.


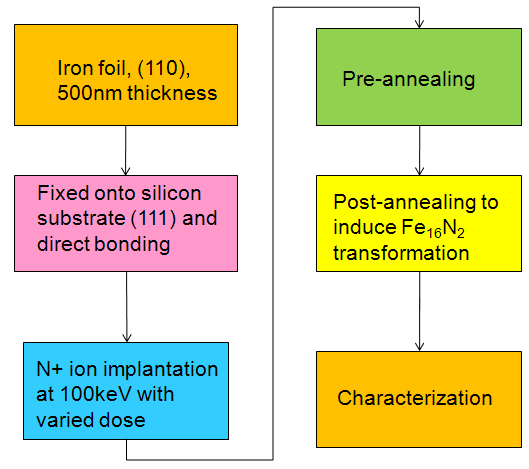


Figure S1 Technical process for sample preparation

In this way, a specific two-step post-annealing process is designed for stress generation and phase transformation. The pre-annealing step is at 500 ºC in a N_2_ and Ar mixture atmosphere for 0.5 hour and the post-annealing step is at 150 ºC for 40 hours in a vacuum. The pre-annealing step activates the implanted nitrogen. The temperature is increased to 500 ºC to initiate the activation and remains at 500 ºC for 0.5 hour to assist the activation over the entire wafer. Besides the activation function, the pre-annealing step also functions to repair the lattice damage incurred at 500 °C in Ar. At the same time, oxygen in the sample could be cleaned in this environment.

Then the post-annealing step is carried out at 150 °C for 40 hrs.

**Auger Electron Spectroscopy (AES) Characterization Results**

Figure S2 shows the nitrogen depth profiles for the ion implanted sample before and after annealing, which are measured by Auger Electron Spectroscopy (AES) with Ar^+^ as the in-depth milling source.

Fig. S2(a) shows the nitrogen distribution before annealing for the four samples with implantation fluences of 2×10^16^ /cm^2^, 8×10^16^ /cm^2^, 1×10^17^ /cm^2^, 5×10^17^ /cm^2^ and 1×10^18^ /cm^2^. The nitrogen implantation range inside the foil is determined by the implantation energy. As shown in Fig. S2, five samples have the same implantation range (160 nm) and same peak position (100 nm). This is coincident with the simulation result by SRIM.

Fig. S2(b) shows the nitrogen depth profiles after the annealing. It can be seen that nitrogen concentrations in the foils are homogeneously distributed inside the whole foils after annealing. The nitrogen concentration distribution corresponding to 1×10^17^ /cm^2^ fluence has reached to 11 at.%, close to the stoichiometric ratio of nitrogen in Fe_16_N_2_. For the sample with 5×10^17^ /cm^2^ and 1×10^18^ /cm^2^, their concentration has surpassed the achievable maximum nitrogen solubility in bcc iron.


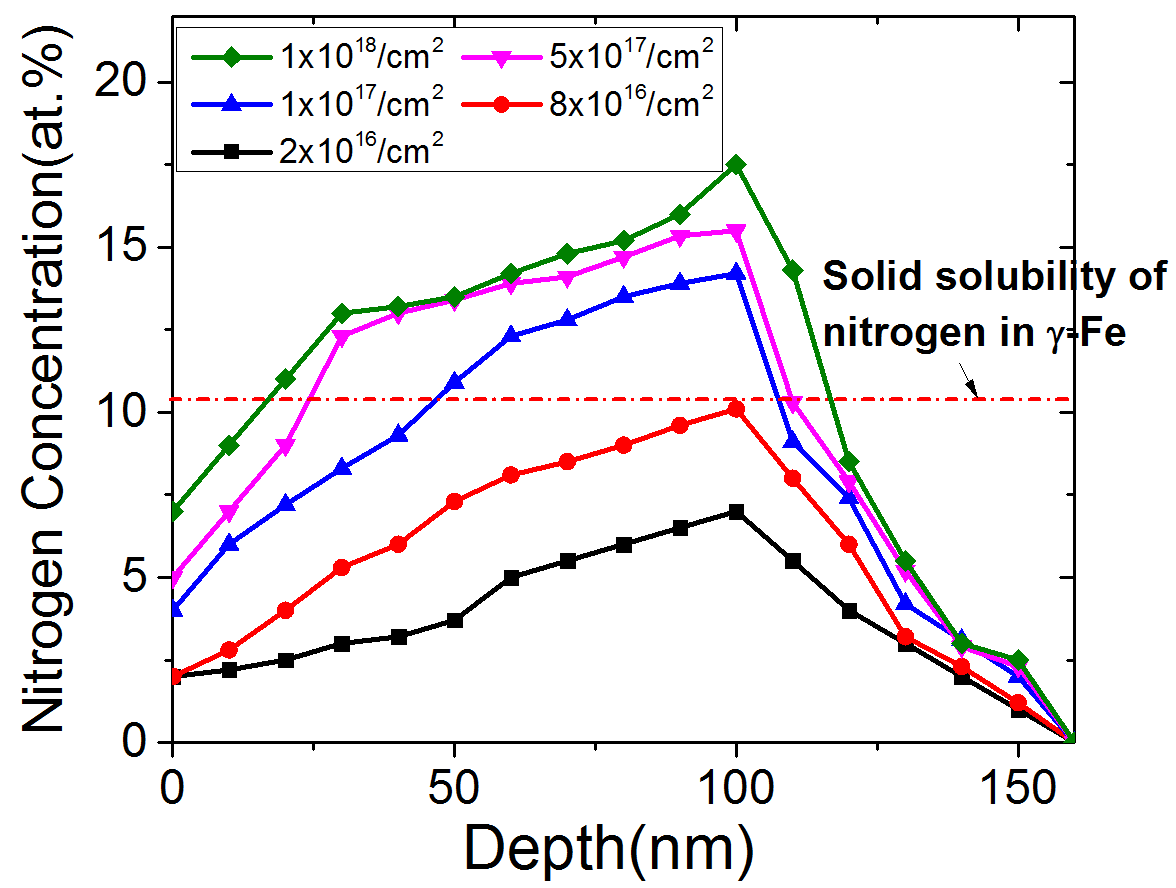

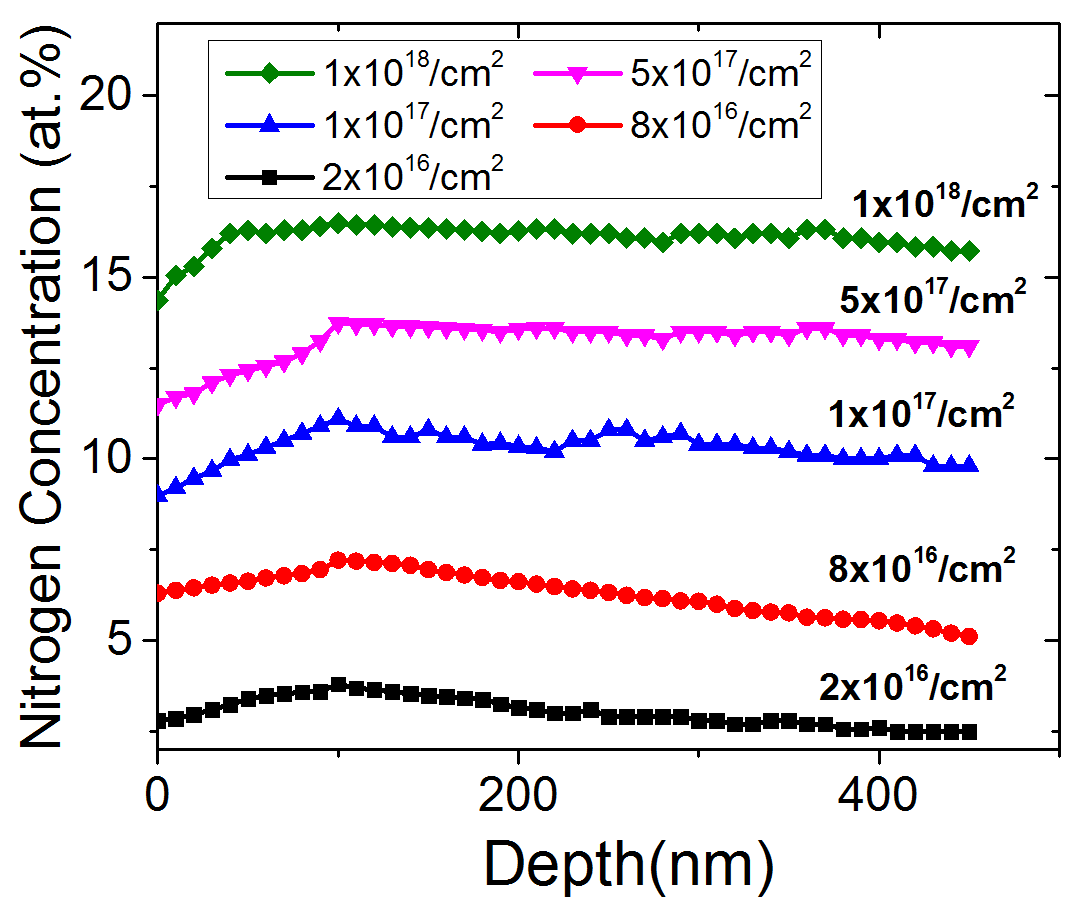


Figure S2(a) In-depth distribution profiles of nitrogen concentration in FeN foils before annealing tested by Auger Electron Spectroscopy (AES), showing that the implant range is 160 nm and peak position at 100 nm.

Figure S2(b) In-depth distribution profiles of nitrogen concentration in FeN foils after annealing tested by (AES), showing nitrogen concentrations are homogeneously distributed inside the whole foils after annealing.

For the sample prepared with 1×10^17^/cm^2^fluence, its nitrogen concentration is almost 11.1 at.% with a homogeneous distribution, corresponding to a maximum volume ratio of α˝ phase[^2^](#_ENREF_2). For the samples prepared with lower doses, including 2×10^16^ /cm^2^ and 8×10^16^ /cm^2^, Fe_16_N_2_ peaks can still be observed after the same annealing procedure.

For the sample prepared with 5×10^17^ /cm^2^ and 1×10^18^ /cm^2^ fluence, its nitrogen concentration is larger than 11.1 at.%, as shown in Fig. S2(b), in the overdoped situation.

**X-ray diffraction (XRD) spectra**

The crystal structure of foil samples is characterized using a Siemens D5005 X-ray diffractometer (XRD) with Cu Kα radiation source. Figure S3 shows the XRD spectra for the foil samples with different nitrogen fluences on Si(111) substrate after post-annealing. It shows that Fe_16_N_2_ phase always exists at all conditions with ion fluences varying from 2×10^16^ /cm^2^ to 1×10^18^ /cm^2^. For ion fluences of 2×10^16^ /cm^2^ and 8×10^16^ /cm^2^, only the Fe_16_N_2_ phase is observed. For the samples with fluences of 1×10^17^ /cm^2^ and 5×10^17^ /cm^2^, because their nitrogen concentrations are larger than 11.1at.%, the ε-iron nitride peak is observed. For samples with fluences of 1×10^18^ /cm^2^, the Fe_16_N_2_ phase is decomposed into Fe_4_N. The FeSi (111) phase can be observed at all the fluences, indicating an iron silicide interface appearing caused by fusion bonding at 450°C[^3^](#_ENREF_3).


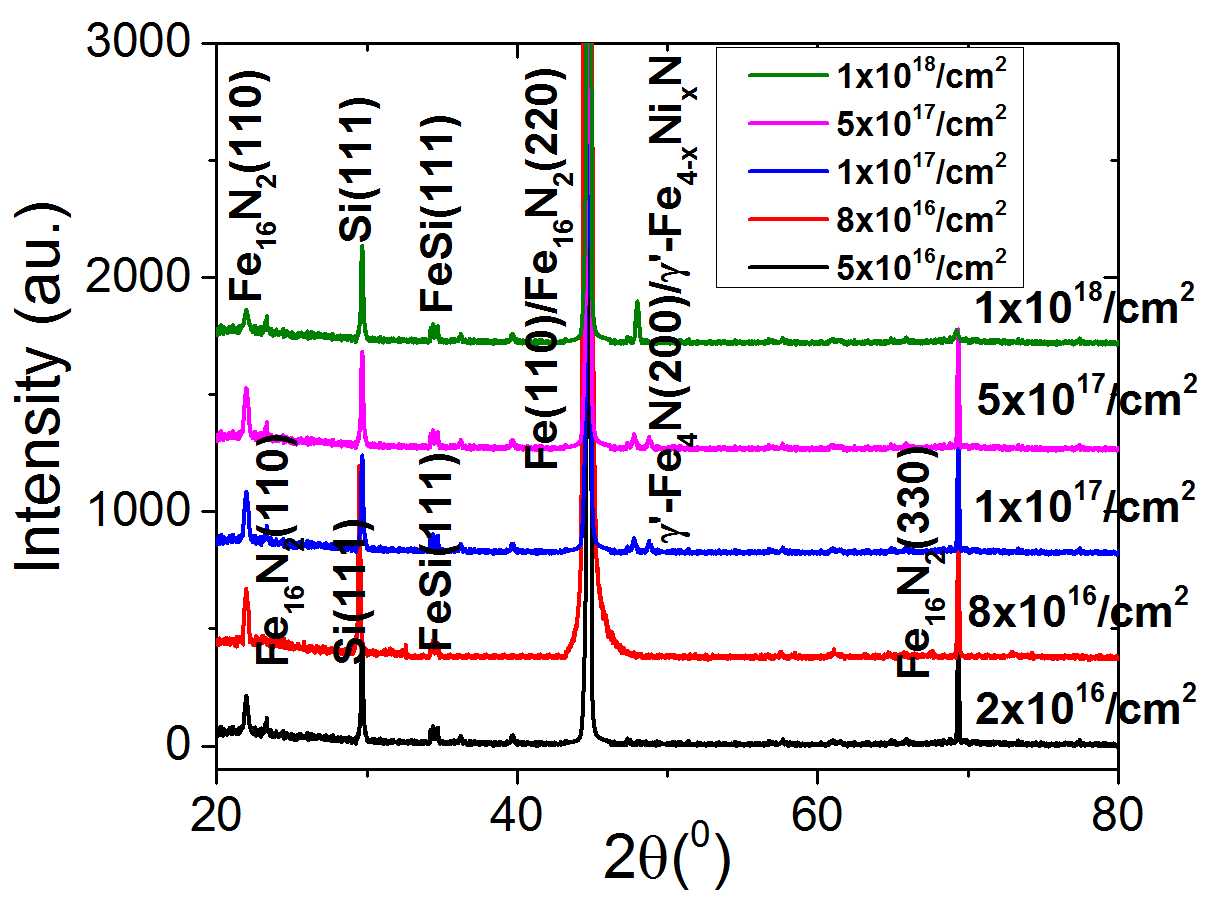


Figure S3 (a). X-Ray diffraction spectra for foil samples with different nitrogen fluences on Si(111) substrate. The spectra from bottom to top correspond to 2×10^16^ /cm^2^, 8×10^16^ /cm^2^, 1×10^17^ /cm^2^, 5×10^17^ /cm^2^,and 1×10^18^ /cm^2^, respectively. For ion fluencies of 2×10^16^ /cm^2^ and 8×10^16^ /cm^2^, only the Fe_16_N_2_ phase was observed. For ion fluencies of 1×10^17^ /cm^2^, 5×10^17^ /cm^2^, and 1×10^18^ /cm^2^, both the ε-iron nitride and Fe_16_N_2_ phase were produced at the same time.


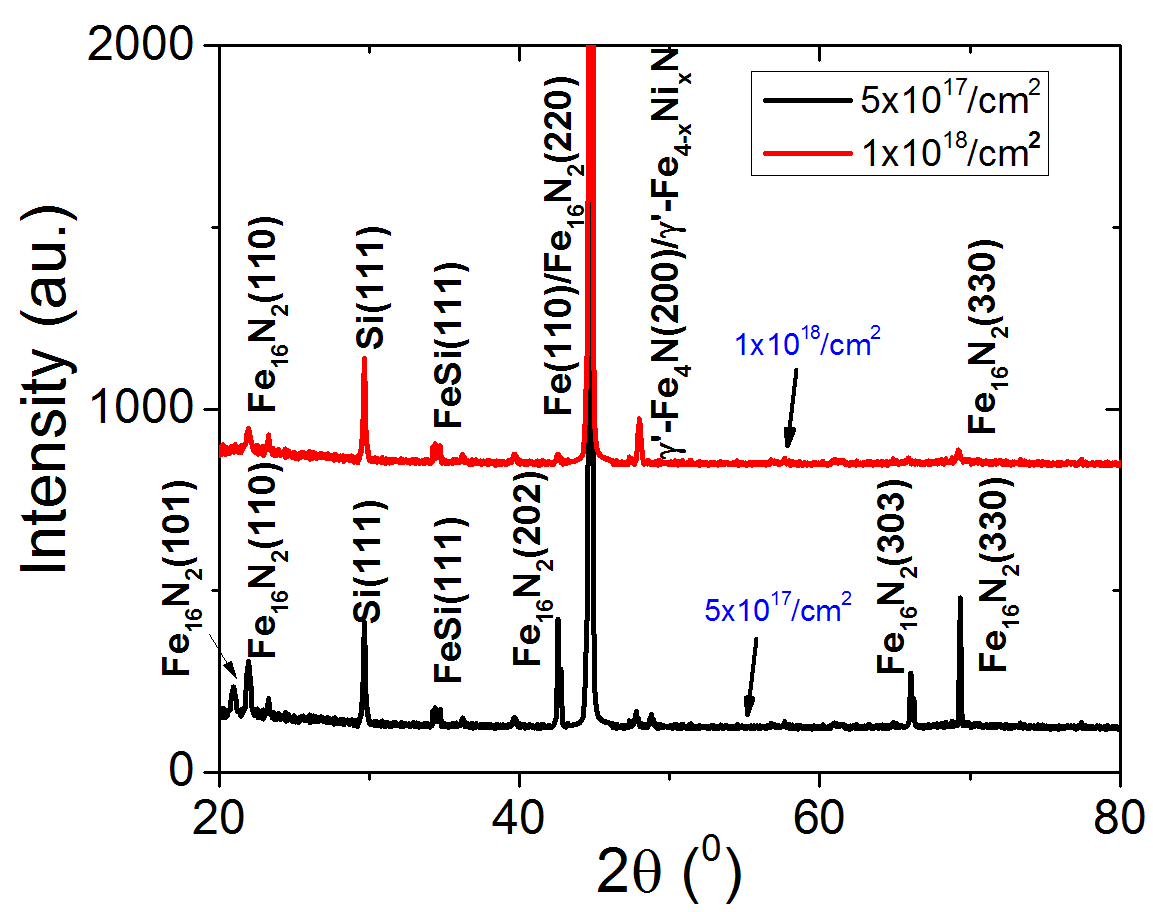


Figure S3 (b). Grazing incident angle x-ray diffraction patterns for the samples with fluencies 5×10^17^ /cm^2^ and 1×10^18^ /cm^2^, respectively. For the sample with fluence 5×10^17^ /cm^2^, Fe_16_N_2_ (101), (202) and (303) peaks can be observable although the intensities are weak compared with the adjacent (hh0) peak because of a strong (hh0) texture on surface. For the sample with fluence 1×10^18^ /cm^2^, no obvious (h0h) peak is observed.

**TEM diffraction pattern**

TEM image of the sample with fluence 5x10^17^/cm^2^ is shown in Figure. S4(a). Its actual diffraction pattern was obtained using the Hitachi HF-3300 TEM, showing in Figure. S4(b). The orientation was set to the same Zone Axis, which shows to be consistent with the <001> zone of Fe_16_N_2_, as shown in Figure. S4(c). The calculated <001> Fe_16_N_2_ diffraction pattern is shown in Figure. S4(d), in which circled spots are “superlattice” reflections in the pattern. It can be seen that the calculated <001> Fe_16_N_2_ diffraction pattern in Figure. S4(d) matches to actual electron diffraction pattern of Figure. S4(b) in a near-perfect manner. This match can demonstrate the existence of α"-Fe_16_N_2_ phase directly.

As shown in Figure. S4(d), the calculated <001> Fe_16_N_2_ diffraction pattern doesn’t show any <h01>. This means that the residual reflections presumably don’t show in TEM for the orientations that have very low intensity in XRD pattern.


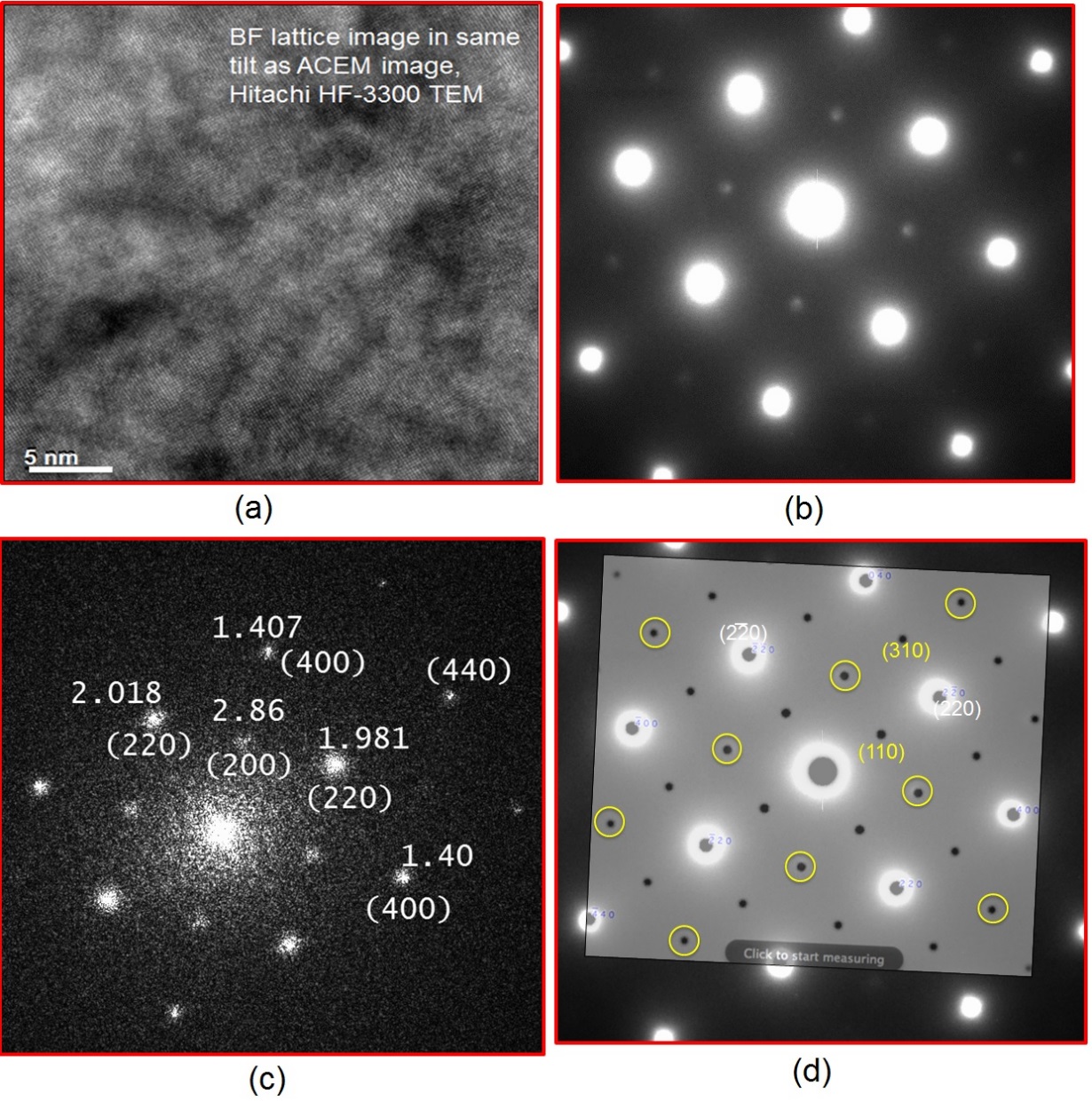


Figure. S4 TEM images and match with calculated α"-Fe_16_N_2_ phase (a) BF lattice image in same tilt as ACEM image by using Hitachi HF-3300 TEM; (b) Actual electron diffraction pattern of same FIB sample; (c) Diffractogram of entire image area shown in (a); (d) Match of calculated <001> Fe_16_N_2_ DP to actual electron diffraction pattern shown in (b). Circled spots are “superlattice” reflections in the pattern.

**Hysteresis loops before ion implantation and before post-annealing**

Figure. S5 shows samples’ hysteresis loops at different stages, including before ion implantation and before post-annealing. For the pure iron foil before ion implantation, its magnetic property is in good agreement with Fe (110) single crystal (loop a). Its remanent magnetization value is equal to its saturation magnetization value with 4πM_S_, which is around 206 emu/g at room temperature. For the sample after the ion-implantation and 500 ºC x 0.5 hr pre-annealing step (loop b), its saturation magnetization increases about 7%, up to 221 emu/g. Meanwhile, its remanent magnetization value is reduced and its saturation field (Hs) is enhanced up to about 1000 Oe, which indicates the existence of the Fe_8_N phase (the stoichiometric Fe_16_N_2_ phase) after the pre-annealing step.

Figure S5 In-plane hysteresis loops for the samples at room temperature (a) before ion-implantation; (b) before post-annealing.

**Strain characterization**

The mismatch of the thermal expansion coefficient between the foil and the substrate generates the compressive strain. Figure S6 shows the strain by comparing XRD spectrum before and after annealing.


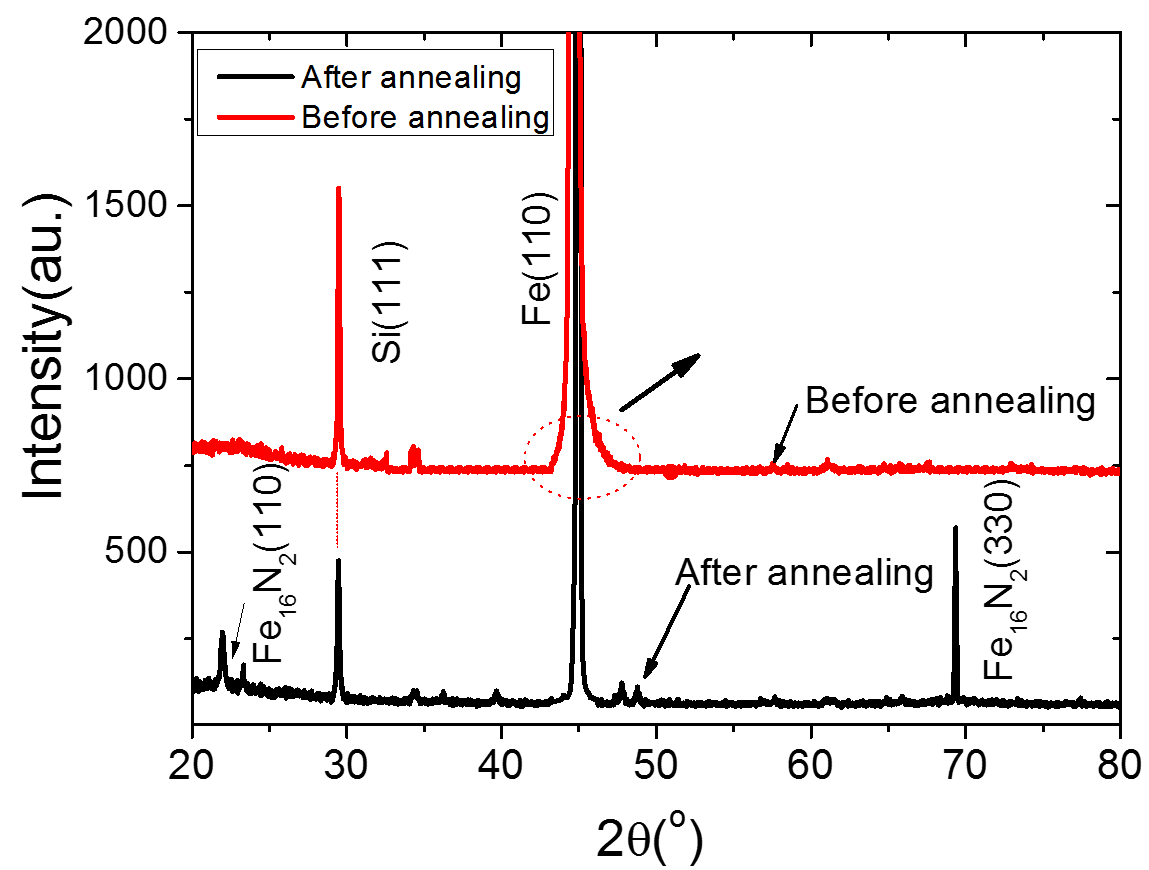

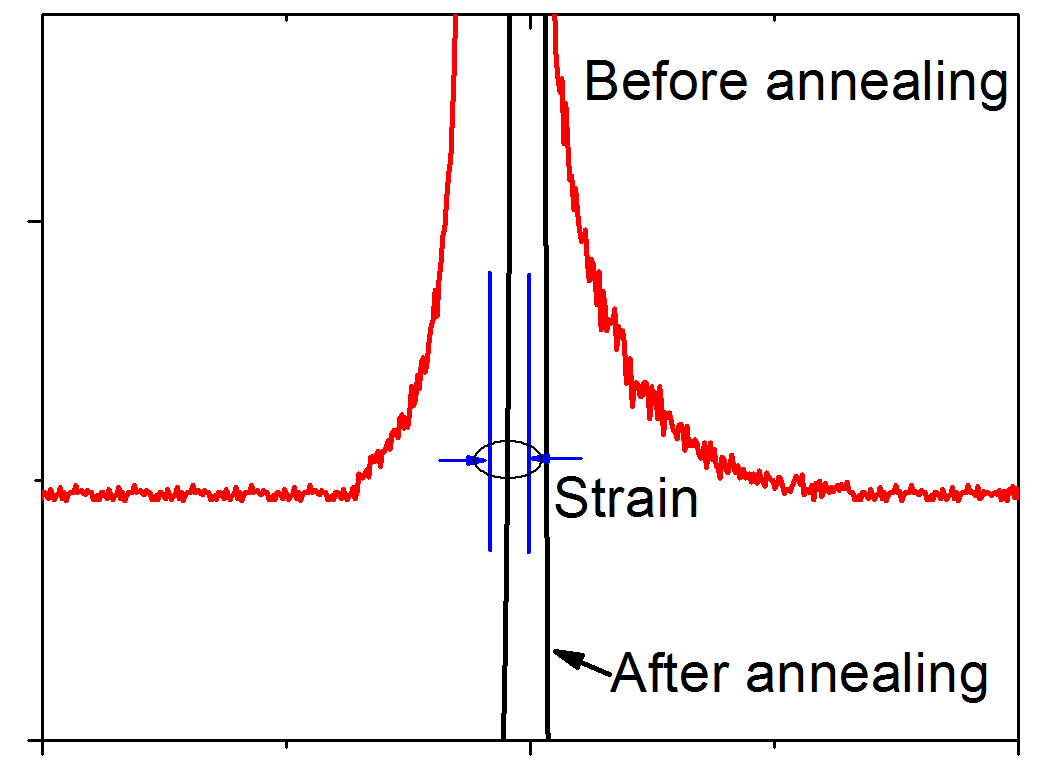


Figure S6 XRD spectra of the ion implanted sample before and after post-annealing. The positions of Si (111) peak before and after annealing remain same, as directed by line. The zoom-in result of dashed circle shows an obvious peak shift, demonstrating the existence of compressive strain in the sample after annealing.

**References**

1 Tong, Q.Y., Cha, G., Gafiteanu, R. & Gosele, U. Low Temperature Wafer Direct Bonding. *J. Microelectromech. Syst.* **3**, 29-35 (1994).

2 Jack, K. H. The occurrence and the crystal structure of α˝-iron nitride; a new type of interstitial alloy formed during the tempering of nitrogen-martensite. *Proc. R. Soc. London. A***208**, 216-224 (1951).

3 Kafader, U., Pirri, C., Wetzel, P. & Gewinner, G. Epitaxial cubic iron silicide formation on Si(111). *Appl. Surf. Sci* **64**, 297-306 (1993).
